# 3D cardiac navigation with rapid multi shot EPI

**DOI:** 10.1186/1532-429X-14-S1-W32

**Published:** 2012-02-01

**Authors:** Aaron T Hess, André J van der Kouwe, Stefan Neubauer, Matthew D Robson

**Affiliations:** 1 Department of Cardiovascular Medicine, John Radcliffe Hospital, University of Oxford Centre for Clinical Magnetic Resonance Research, Oxford, UK; 2 Athinoula A. Martinos Center for Biomedical Imaging, MGH, Charlestown, MA, USA

## Summary

To assess a rapid 3D multishot EPI acquisition as an improved cardiac respiratory navigator.

## Background

3D EPI navigators are a robust real-time brain navigation tool [1], they allow rapid online reconstruction and image registration (< 80 ms). A thoracic EPI volume can be acquired in 200 ms, thus allowing real-time navigation. An analysis of the EPI navigators’ stability and variance when registering the heart is presented.

## Methods

EPI parameters were: flip angle 2°, FOV (v1) 332 x 221 x 144 mm^3^ or (v2 to v4) 400 x 300 x 150 mm^3^, acquisition matrix 48 x 36 x 18, TR 14 ms, TE 6.3 ms, slice partial Fourier 6/8, and bandwidth 3858 Hz/pixel, acquisition time 200 ms. The registration region of interest (ROI), the heart, was identified using the adjustment volume. The images were reconstructed in real-time and fed into a modified 3D PACE rigid body registration [2] which registered the ROI to that of the first navigator’s volume.

Four volunteers (mean age 32 +/- 7 years) were scanned on a Siemens 3T. For each, a scan was acquired with 50 navigator volumes, one per R-R interval. Each volunteer held their breath at end expiration for +/- 10 heart beats, then at end inspiration for +/- 10 heart beats, repeating this until the end of the scan. A fifth volunteer was instructed to breathe deeply for the entire scan. Finally the navigators’ impact on Mz was measured with a Bloch simulator [3].

## Results

A sample navigator volume and the translations and rotation estimates from one volunteer are shown in the figure. The standard deviation of each motion estimate, calculated as in [4] and by excluding transitions zones, are presented in the table. These measures demonstrate an upper limit on registration variance/stability of 0.6 mm and 0.5°. The motion estimates for the fifth volunteer, with deep breathing, exceeded 4 mm or 4° in all measures. The Bloch simulator shows that the sum effect of the 2° flip angles reduces the Mz by 0.7%.

**Figure 1:**
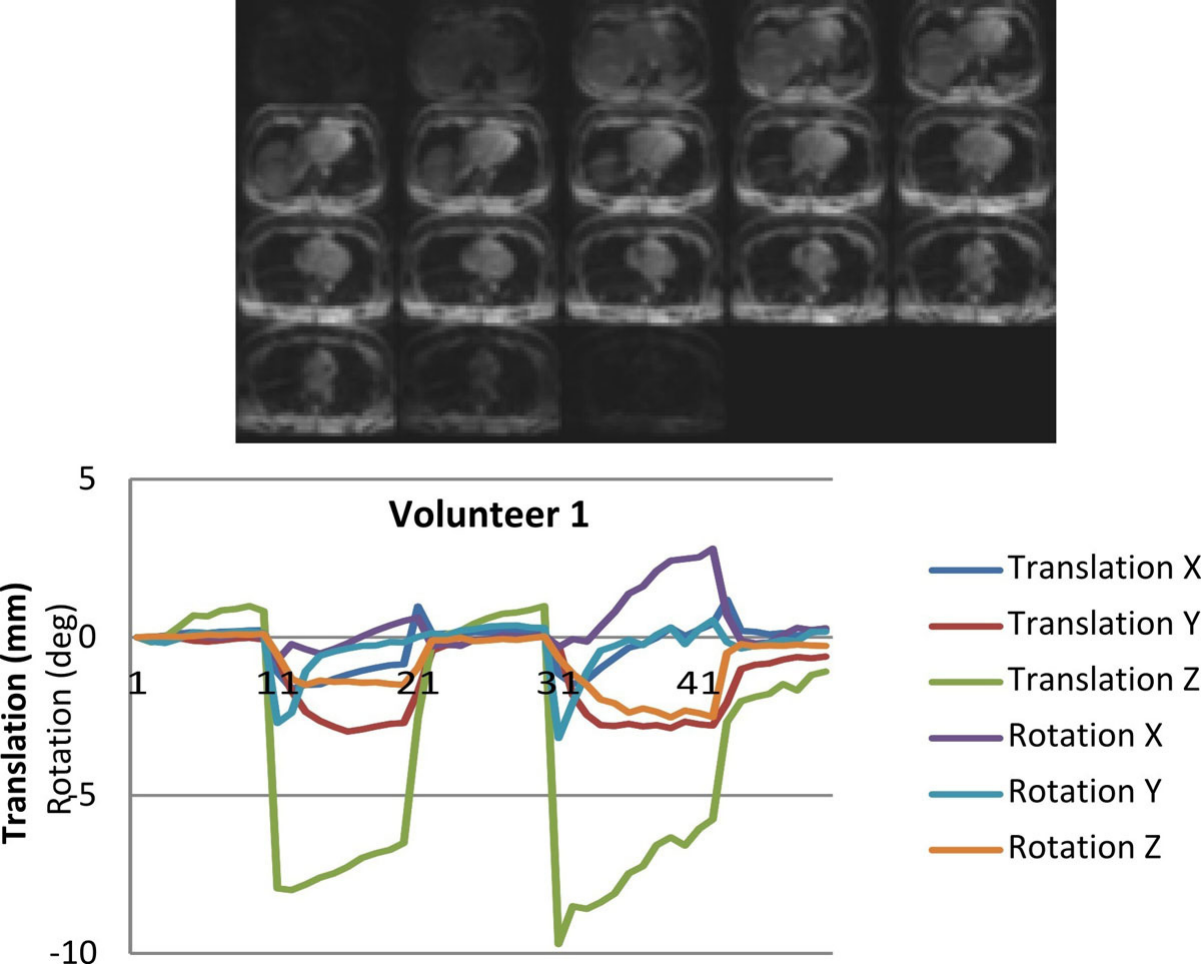
Example navigator volume and registration result from a volunteer

**Table 1:** Standard deviation of registration during breathhold periods

Volunteer	Translation (mm)			Rotation (deg)		
	
	X	Y	Z	X	Y	Z
1	0.3	0.3	0.4	0.5	0.4	0.3
2	0.5	0.3	0.5	0.4	0.4	0.2
3	0.3	0.3	0.6	0.2	0.5	0.3
4	0.6	0.3	0.6	0.2	0.2	0.2

## Conclusions

EPI proves to be rapid, reliable and consistent as a heart navigator. Its 2° flip angle has a minimal effect on the image contrast (Mz). The real-time nature of this navigator would prove particularly beneficial for techniques like spectroscopy, high resolution imaging, and various forms of functional cardiac imaging.

## Funding

This work was supported by the Medical Research Council [G0900883].
